# Cell death-related signature genes: risk-predictive biomarkers and potential therapeutic targets in severe sepsis

**DOI:** 10.3389/fmed.2025.1577203

**Published:** 2025-05-30

**Authors:** Yanan Li, Yuqiu Tan, Zengwen Ma, Weiwei Qian

**Affiliations:** ^1^Department of Emergency, Shangjinnanfu Hospital, West China Hospital, Sichuan University, Chengdu, Sichuan, China; ^2^Laboratory of Emergency Medicine, Department of Emergency Medicine, West China Hospital, and Disaster Medical Center, Sichuan University, Chengdu, Sichuan, China

**Keywords:** sepsis, sepsis-associated acute lung injury or acute respiratory distress syndrome, cell death, signature genes, biomarkers, therapeutic targets

## Abstract

Sepsis is a systemic inflammatory response syndrome that predisposes to severe lung infections (SeALAR) such as sepsis-associated acute lung injury (Se/ALI) or sepsis-associated acute respiratory distress syndrome (Se/ARDS). Through a systematic bioinformatics approach, this study aimed to unravel the pathogenesis of SeALAR and explore potential biomarkers and individualized therapeutic targets. We analyzed differential genes in the peripheral blood of SeALAR patients based on the GSE10474 and GSE32707 datasets, and identified 352 significantly differentially expressed genes. Various signaling pathways related to immune regulation were found to be significantly altered via GO and KEGG enrichment analysis. Further combining cell death-related gene screening and four machine learning algorithms (including LASSO-logistic, Gradient Boosting Machine, Random Forest and xGBoost), nine SeALAR-characterized cell death genes (SeDGs) were screened and a risk prediction model based on SeDGs was constructed that demonstrated good prediction performance. In immunoassays, ssGSEA showed that Activated.CD8.T.cell, CD56bright.natural.killer.cell, MDSC, Natural.killer.T.cell, T.follicular.helper. cell and TType.1.T.helper.cell had significantly lower infiltration abundance than lower infiltration levels compared to the Se group. GSEA analysis revealed key immune pathways in which SeDGs may be involved. In addition, unsupervised clustering analysis revealed that SeALAR patients could be classified into two molecular subtypes, providing a new direction for the development of individualized immunotherapy strategies. In conclusion, this study systematically analyzed the molecular features and immune disorder mechanism of SeALAR from a multidimensional perspective, and thus provides a theoretical basis and potential targets for precision medicine intervention and targeted drug development.

## 1 Introduction

Sepsis is one of the most common complications in intensive care units (ICUs) (1), which usually leads to organ infections, particularly in the lungs, thereby causing acute lung injury (ALI) or acute respiratory distress syndrome (ARDS) due to its complex molecular basis (2, 3). Additionally, ALI often deteriorates and progresses to ARDS (4, 5). Statistically, sepsis-associated ARDS accounts for approximately 32% of all sepsis cases, with an extremely high mortality rate (6, 7). Nevertheless, the presentations of sepsis are highly heterogeneous, which limits the predictive value of single factors (8). Therefore, it is helpful for assessing the severity and prognosis of sepsis to develop new risk pre-diction models for sepsis patients with lung co-infections, such as ALI or ARDS (SeALAR).

During pulmonary infections in septic patients, insufficient oxygen supply pre-disposes to incomplete oxidative reactions or hypoxia, resulting in a sharp increase in free radicals and severe damage to the antioxidant system (1, 9). In such patients, activated peripheral blood cells (especially neutrophils) release inflammatory cytokines, such as tumor necrosis factor-α (TNF-α), interleukin (IL)-1, and chemokines, when exposed to damage-associated molecular patterns (DAMPs) or pathogen-associated molecular patterns (PAMPs) (10–12), eventually inducing many types of cell death, such as apoptosis, pyroptosis, and ferroptosis (13–15). In turn, the dead cells further drive the release and activation of various pro-inflammatory cytokines (including TNF-α, IL-1, IL-2, IL-6, and IL-8) and systemic coagulation abnormalities, thus accelerating the development of the disease, triggering systemic inflammatory cascade reactions and immune disorder, and aggravating lung injury, which contributes to the dismal prognosis of patients (16–20). Accordingly, cell death-related genes may serve as biomarkers and risk and prognostic factors for SeALAR.

In this study, bioinformatics analysis was performed to determine the expression of cell death-related genes in the peripheral blood during the progression of sepsis, and various statistical analysis methods were utilized to screen reliable cell death-related signature genes (SeDGs) in SeALAR. Subsequently, a risk prediction model for SeALAR was constructed based on these genes, followed by immune infiltration analysis and Gene Set Enrichment Analysis (GSEA). Overall, this study aimed to provide new di-rections for future research, diagnosis, and treatment of sepsis.

## 2 Materials and methods

### 2.1 Data source

Two gene expression datasets, GSE10474 (21) and GSE32707 (22), were downloaded from the Gene Expression Omnibus database, followed by the collection of peripheral blood gene expression data of 31 septic patients complicated by ARDS, 13 septic patients complicated by ALI, and 79 septic patients without complications (Se). Inclusion criteria for participants were as follows: SeALAR patients and Se patients. Exclusion criteria for participants were as follows: patients with other diseases complicated by ARDS or ALI. As all data were obtained from public datasets, no additional ethical re-view was required.

### 2.2 Differentially expressed gene (DEG) screening

First, the annotation files of each platform were used to map the probes to the HGNC gene symbols, and after excluding the probes mapped to multiple genes or no corresponding genes, the two sets of matrices were merged based on the unified gene symbols. In order to eliminate the systematic bias between different platforms and batches, the merged matrices for batch effects were corrected using the ComBat function in the sva package, and verified the significant elimination of batch clustering between samples after correction by principal component analysis (PCA). After completing the batch correction, background correction, quantile normalization and log2 transformation were performed on the data in the limma package. Afterward, the design matrix was constructed using the 79 sepsis control cases as a reference, and the lmFit and eBayes functions were called to identify the differentially expressed genes. Here, the screening criterion was set to be FDR < 0.05 and |log2FC| > 0.263. Finally, the filtered DEGs were imported into the clusterProfiler package, and KEGG pathway and GO bioprocess enrichment analysis were carried out. The result was considered significant at the FDR of less than 0.05.

### 2.3 Construction of a risk prediction model based on cell death-related signature genes in SeALAR (SeDGs)

A total of 2,856 cell death-related genes were screened from 18 cell death types reported in the literature (23, 24) (Supplementary Table 1). These cell death genes were then intersected with the DEGs obtained in the previous step using a Venn diagram to yield the cell death-related genes that were differentially expressed in the peripheral blood of SeALAR patients.

To further screen SeALAR-characterized cell death genes (SeDGs) with predictive ability, four commonly used machine learning algorithms are introduced in this study, including LASSO-logistic (25), Gradient Boosting Machine (GBM) (26), Random Forest (RF) (27) and the xGBoost (28) algorithms. Among them, the LASSO-logistic model used 10-fold cross-validation to determine the optimal regularization parameter (lambda), and the regression model was built by the “glmnet” package and the variables are screened; the GBM model was constructed based on the “gbm” package, and the main parameters were set to “gbm.” The GBM model was constructed based on the “gbm” package, and the main parameters were set as interaction.depth = 3, n.trees = 100, shrinkage = 0.1, and cv.folds = 10; the RF model was constructed using the “randomForest” package, and ntree = 500, and mtry = 10. The RF model uses the “randomForest” package, with ntree = 500 and mtry = 500, and takes p to rank the importance of the variables; the xGBoost model was accomplished with the “xgboost” package, with the main parameters max_depth = 6, eta = 0.3, nrounds = 100, and the objective set to “binary: logistic,” and 10-fold cross-validation was also used to improve the stability of the model. Finally, the high-weighted genes with scores greater than five were, respectively extracted from each model, and their intersection was taken to obtain the most stable and consistent characteristic gene set.

Based on the SeDGs obtained from the screening, the “rms” R package was further used to construct a nomogram model for predicting the risk of SeALAR. The consistency between the predicted and actual values of the model was assessed by plotting a calibration curve, and the Net Benefit of the model under different risk thresholds was evaluated by Decision Curve Analysis (DCA). All statistical tests were considered statistically significant when the *p*-value was less than 0.05.

### 2.4 Immune infiltration analysis, GSEA, and clustering analysis based on SeDGs

Correlations of SeDGs with the degree of immune cell infiltration were analyzed with “ggstatsplot” and “ggplot2” software packages. Afterward, the enriched pathways of each SeDG were identified with GSEA software. Then, all SeALAR patients were subjected to cluster analysis with the “ConensusClustosPlus” software package, followed by the drawing of principle component analysis (PCA) curves, as well as ex-pression heatmaps and box plots of SeDGs. The results were statistically significant when the P value was less than 0.05.

### 2.5 Statistical analysis

R (version 4.1.0) was used for statistical analysis. Differences were considered statistically significant at *P* < 0.05.

## 3 Results

### 3.1 Differential gene expression profiling and pathway enrichment analysis of peripheral blood from SeALAR patients

**FIGURE 1:**
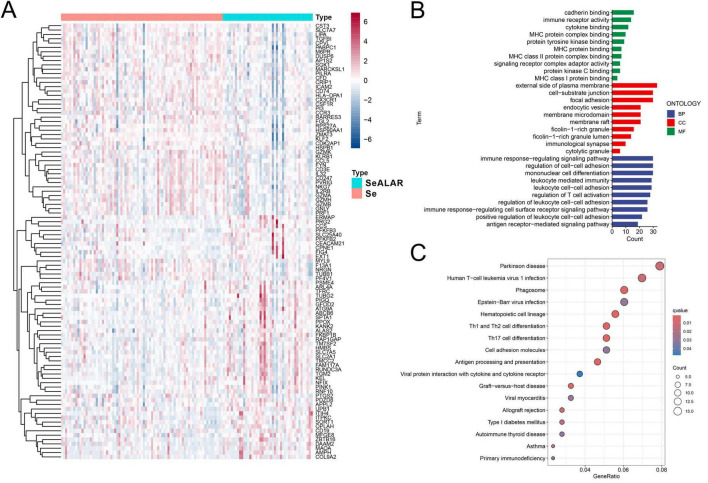
Differential gene screening and GO and KEGG enrichment analyses. **(A)** A heatmap demonstrating the top 100 genes with the most significant differences in the peripheral blood of sepsis is a systemic inflammatory response syndrome that predisposes to severe lung infections (SeALAR) patients with lung infections (*n* = 44) versus septic patients (*n* = 79), with adjusted false discovery rate (FDR) < 0.05 and |log2FC| > 0.263 as the screening criteria for differential genes. **(B)** GO functional enrichment analysis of differential genes, where the results are statistically significant when FDR is less than 0.05. **(C)** KEGG pathway enrichment analysis of differential genes, where the results are statistically significant when FDR is less than 0.05.

Initially, changes in peripheral blood gene expression profiles during the progression of sepsis to ALI or ARDS were analyzed with the limma package, which yielded 352 DEGs, including 91 upregulated genes and 261 downregulated genes (Supplementary Table 2). The heatmap shows the top 100 genes with the most significant differences (Figure 1A).

To delve into the underlying molecular mechanisms in the peripheral blood when Se developed into SeALAR, the obtained DEGs were subjected to GO and KEGG pathway enrichment analyses. The results of the GO functional enrichment analysis displayed that immunoregulation-related pathways were markedly altered in the peripheral blood of SeALAR patients (Figure 1B and Supplementary Table 3). In addition, the results of the KEGG pathway enrichment analysis revealed significant enrichment of Parkinson’s disease, human T-cell leukemia virus type 1 infection, phagosomes, Epstein-Barr virus infection, and Th1 and Th2 cell differentiation in SeALAR (Figure 1C and Supplementary Table 4). These results indicate that SeALAR may occur as a result of immune abnormalities and may trigger other system diseases.

### 3.2 Identification of SeDGs

To screen reliable cell death-related biomarkers in SeALAR, DEGs were intersected with previously identified cell death-related genes to obtain 108 cell death-related genes differentially expressed in SeALAR (Supplementary Figure 1 and Supplementary Table 5). Sub-sequently, these 108 genes were analyzed by GBM, xGBoost, LASSO-logistic, and RF algorithms to screen SeDGs.

**FIGURE 2:**
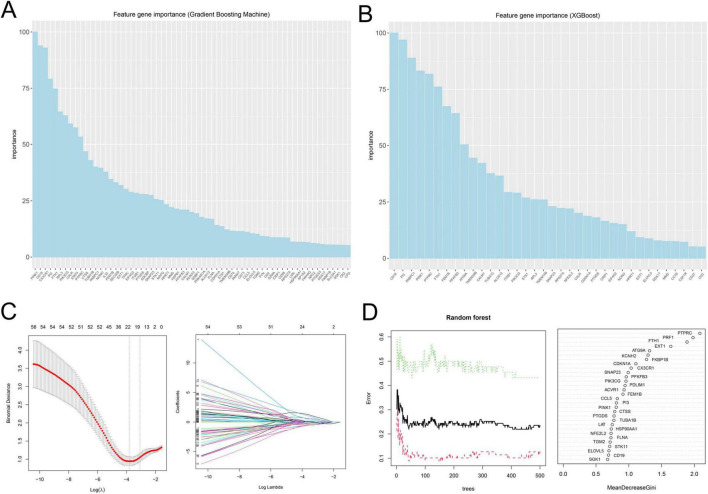
Signature genes in sepsis is a systemic inflammatory response syndrome that predisposes to severe lung infections (SeALAR) identified by four machine learning algorithms. **(A)** A total of 63 signature genes identified by GBM. **(B)** A total of 36 signature genes identified by xGBoost. **(C)** A total of 22 signature genes identified by LASSO-logistic. **(D)** A total of 30 signature genes identified by Random Forest (RF). The results are statistically significant when P is less than 0.05.

**FIGURE 3:**
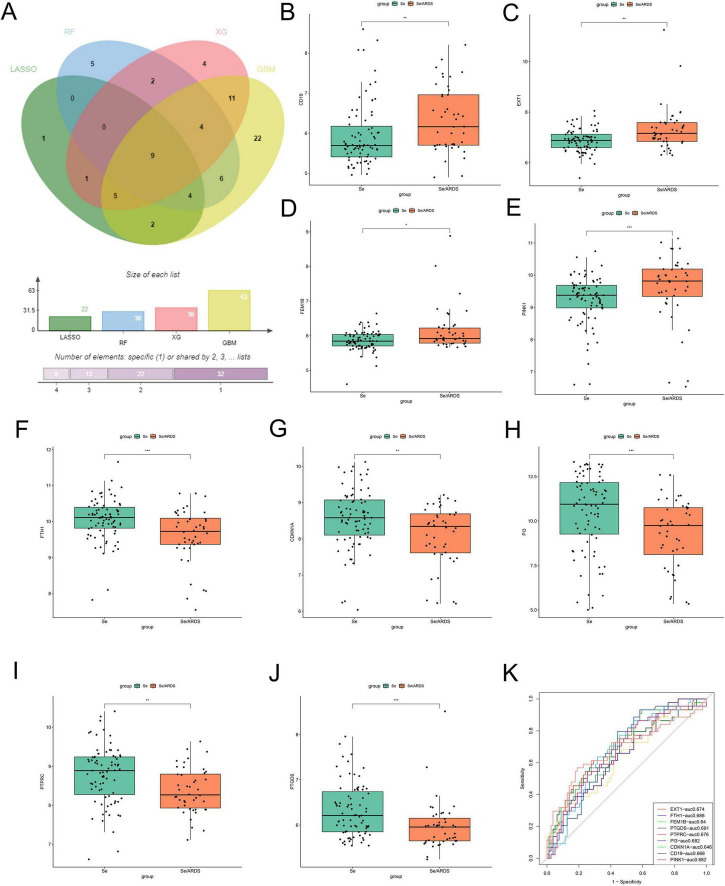
Screening and expression analysis of characterized genes in the peripheral blood of patients with sepsis is a systemic inflammatory response syndrome that predisposes to severe lung infections (SeALAR) and simple sepsis. **(A)** Venn diagram showing the nine SeALAR characterized genes jointly identified by four machine learning algorithms, namely LASSO, Random Forest (RF), Gradient Boosting Machine (GBM) and eXtreme Gradient Boosting (XGBoost). The box-and-line plots show the differential expression of the nine characterized genes between the se/ARDS group and the simple sepsis group **(B–J)**. Among them, CD19 **(B)**, EXT1 **(C)**, FEM1B **(D)**, and PINK1 **(E)** were significantly highly expressed in the peripheral blood of patients in the se/ARDS group; and CDKN1A **(F)**, FTH1 **(G)**, PI3 **(H)**, PTGDS **(I)**, and PTPRC **(J)** were significantly less expressed in the peripheral blood of patients in the se/ARDS group. Statistical analysis was performed using Wilcoxon test with significance levels marked as *P < 0.05, **P < 0.01, ***P < 0.001. **(K)** ROC curves were analyzed for the diagnostic value of the nine characteristic genes used to differentiate between se/ARDS and simple sepsis, and the corresponding area under the curve (AUC) values were labeled separately.

The results demonstrated that there were 63 signature genes identified by GBM (Figure 2A), 36 signature genes identified by xGBoost (Figure 2B), 22 signature genes identified by LASSO-logistic (Figure 2C), and 30 signature genes identified by RF (Figure 2D). To attain more precise SeDGs, the signature genes identified by these algorithms were intersected. Eventually, nine reliable SeDGs were determined (Figure 3A), among which CD19, EXT1, FEM1B, and PINK1 were upregulated (Figures 3B–E) and FTH1, CDKN1A, PI3, PTPRC, and PTGDS were downregulated (Figures 3F–J) in the peripheral blood of SeALAR patients. The area under the receiver-operating characteristic curve further confirmed the accuracy of these nine SeDGs as biomarkers in SeALAR (Figure 3K).

### 3.3 Construction of a SeDG-based risk prediction model for SeALAR

**FIGURE 4:**
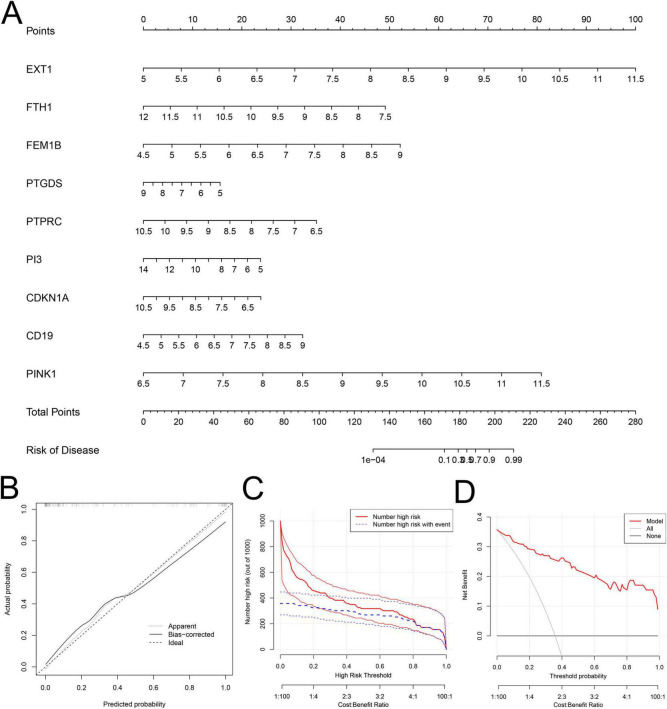
Construction and evaluation of a clinical risk prediction model based on sepsis is a systemic inflammatory response syndrome that predisposes to severe lung infections (SeALAR) signature genes. **(A)** Nomograms of nine genes (EXT1, FTH1, FEM1B, PTGDS, PTPRC, PI3, CDKN1A, CD19, and PINK1) were constructed to predict the risk of sepsis secondary to lung infection. Corresponding scores were obtained from the expression levels of each gene, and the total score was obtained by summing up all gene scores to predict the risk probability of developing SeALAR in Se patients. **(B)** Calibration curve analysis shows the agreement between the model predicted probabilities and the actual observed probabilities to assess the accuracy of the prediction model. **(C,D)** Decision Curve Analysis (DCA) was used to assess the clinical benefit and utility of the predictive model. **(C)** Shows the number of patients predicted to be at high risk by the model at different risk thresholds and the number of patients who actually develop the disease; **(D)** Shows the Net Benefit of applying the model to clinical decision-making at different threshold probabilities.

Due to the high mortality rate of SeALAR, constructing a risk prediction model for SeALAR is clinically important to improve the prognosis of septic patients. In this study, the obtained SeDGs were utilized to construct a risk prediction model for SeALAR, which was represented as a nomogram. Through the detection of SeDG expression in peripheral blood, the risk of SeALAR could be assessed, providing prognostic information for septic patients (Figure 4A). The results of calibration curves and DCA verified that the SeDG-based nomogram model had superior clinical prediction performance (Figures 4B–D).

### 3.4 Immune correlation analysis of SeALAR

**FIGURE 5:**
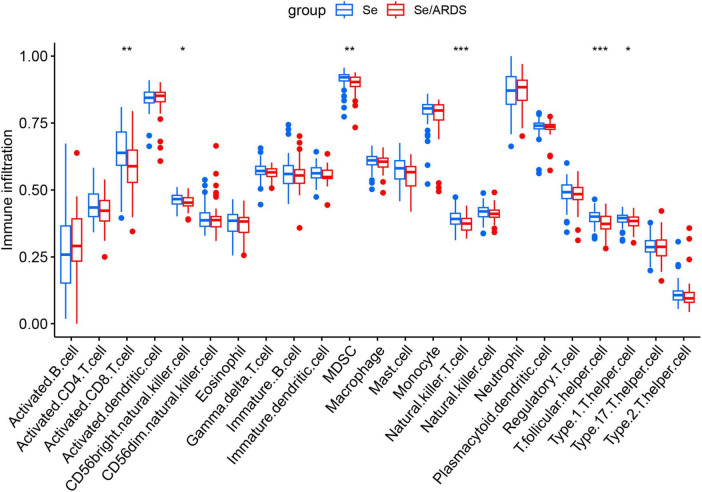
Characterization of immune cell infiltration in peripheral blood of patients with sepsis-associated acute respiratory distress syndrome (Se/ARDS) versus simple sepsis. Levels of infiltration of 28 immune cell types in the peripheral blood of the two groups of patients were assessed based on single-sample Gene Set Enrichment Analysis (ssGSEA). The statistical method included Wilcoxon rank-sum test, and the significance levels were expressed as *P < 0.05, **P < 0.01, and ***P < 0.001.

Previous studies have demonstrated that the immune function of SeALAR patients is highly disturbed and that immunity deficiency usually results in a poor prognosis for patients (1, 29). Therefore, it is of clinical significance to analyze the changes in the immune microenvironment of SeALAR for improving the prognosis of SeALAR. In this study, the infiltration abundance of immune cells was calculated with ssGSEA. Preliminary results exhibited lower infiltration abundances of Activated.CD8.T.cell, CD56bright.natural.killer.cell, MDSC Natural.killer.T.cell, T.follicular.helper.cell and Type.1.T.helper.cell in the peripheral blood of Se/ARDS patients than in the peripheral blood of Se patients (Figure 5). Further, the correlation between SeDGs and immune-infiltrating cells was analyzed with the “ggstatsplot” and “ggplot2” software packages, providing potential targets for personalized immunotherapy.

**FIGURE 6:**
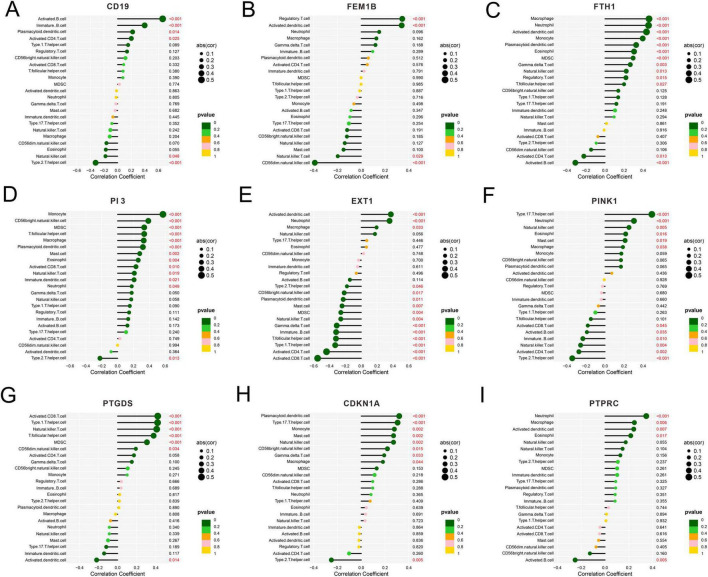
Correlation analysis between sepsis is a systemic inflammatory response syndrome that predisposes to severe lung infections (SeALAR) characterized genes and immune cell infiltration. **(A–I)** Spearman’s correlation between the expression levels of nine characterized genes (CD19, FEM1B, FTH1, PI3, EXT1, PINK1, PTGDS, CDKN1A, PTPRC) and the abundance of immune cell infiltration was assessed based on the ssGSEA method. The horizontal coordinate indicates the Correlation Coefficient, the size of the point represents the absolute value of the correlation (|cor|), and the color indicates the size of the *P*-value.

The results manifested the presence of a correlation between CD19 and activated B cells (Figure 6A), between FEM1B and regulatory T cells (Figure 6B), between FTH1 and macrophages (Figure 6C), between PI3 and monocytes (Figure 6D), between EXT1 and activated dendritic cells (Figure 6E), between PINK1 and T helper 17 cells (Figure 6F), between PTGDS and activated CD8 T cells (Figure 6G), and between CDKN1A and plasmacytoid dendritic cells (Figure 6H). In addition, PTPRC had the highest correlation coefficient with neutrophils (Figure 6I). Accordingly, in different SeALAR patients, specific targets are needed for treatment against different immune cell changes.

### 3.5 GSEA results

**FIGURE 7:**
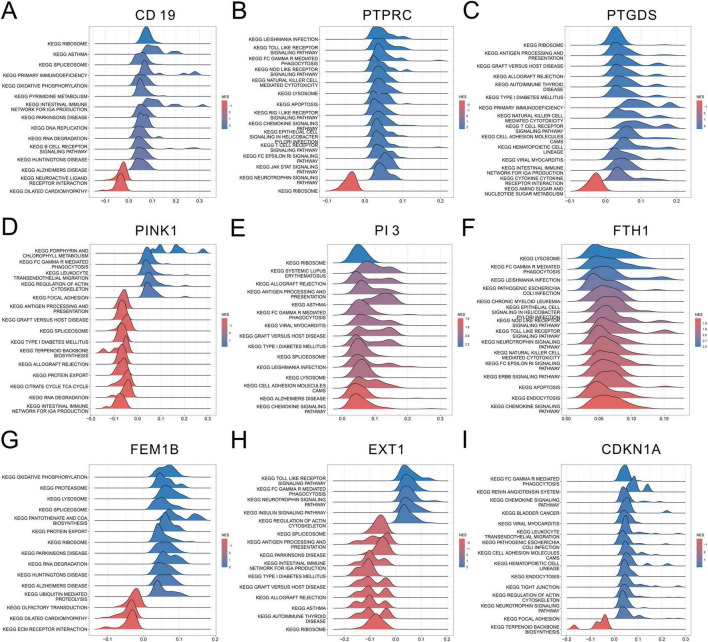
Gene Set Enrichment Analysis (GSEA) analysis of sepsis is a systemic inflammatory response syndrome that predisposes to severe lung infections (SeALAR) characterized genes. **(A–I)** GSEA was used to explore the nine characterized genes (CD19, PTPRC, PTGDS, PINK1, PI3, FTH1, FEM1B, EXT1, and CDKN1A) in different KEGG pathway Potential functions. The figure demonstrates the pathway significance enrichment associated with high expression of each gene, the horizontal coordinate is the enrichment score (NES), and the color indicates the enrichment direction.

Gene Set Enrichment Analysis was performed further to investigate the role of each SeDG in SeALAR. The results displayed that CD19 (Figure 7A), PTPRC (Figure 7B), PTGDS (Figure 7C), PINK1 (Figure 7D), PI3 (Figure 7E), FTH1 (Figure 7F), FEM1B (Figure 7G), EXT1 (Figure 7H), and CDKN1A (Figure 7I) were mainly enriched in the RIBOSOME pathway, the LEISHMANIA INFECTION pathway, the RIBOSOME pathway, the PORPHYRIN AND CHLOROPHYLL METABOLS pathway, the RIBOSOME pathway, the LYSO-SOME pathway, the OXIDATIVE PHOSPHORYLATION pathway, the TOLL LIKE RECEPTOR pathway, and the FC GAMMA R MEDIATED PHAGOCYTOSIS pathway, respectively. Furthermore, SeDGs and their 20 interacting genes were subjected to protein-protein interaction analysis with the GeneMANIA database, which clarified the potential regulatory mechanisms of SeDGs (Supplementary Figure 2). These results preliminarily disclosed the molecular mechanism of SeDGs and provided theoretical guidance for the targeted therapy of SeALAR.

### 3.6 Cluster analysis of SeALAR

**FIGURE 8:**
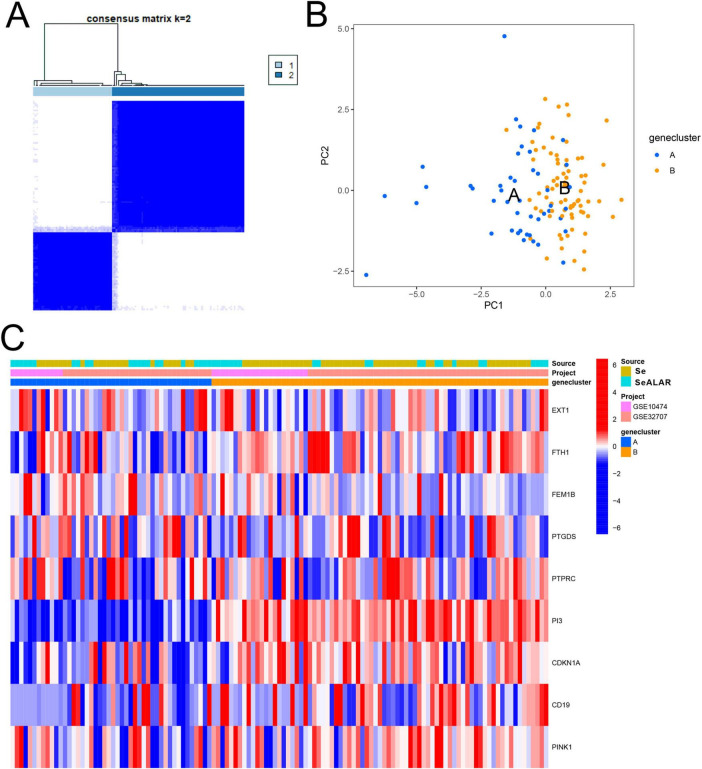
Molecular subtyping analysis based on sepsis is a systemic inflammatory response syndrome that predisposes to severe lung infections (SeALAR) signature genes. **(A)** The results of the consensus clustering analysis showed that patients with sepsis could be stably classified into two molecular subtypes (Cluster 1 and Cluster 2) at the number of clusters K = 2. The consensus matrix heat map in the figure shows the consistency of clustering between samples. **(B)** Principal component analysis (PCA) further validated the above clustering results, showing that the characteristic genes can clearly distinguish patients into two subtypes [gene clusters A and B) with good discriminatory ability. **(C)** Heatmap demonstrating the expression distribution of nine characteristic genes (EXT1, FTH1, FEM1B, PTGDS, PTPRC, PI3, CDKN1A, CD19, PINK1) in different subtypes (A vs. B) as well as in different groups (Se vs. Se/ARDS), with the sample source (GSE10474 vs. GSE32707) and the typing categories. Red color indicates high gene expression and blue color indicates low expression.

Subsequently, a cluster analysis was performed on all SeALAR patients with the “ConensusClustosPlus” software package based on the above SeDGs. It was found that when K = 2, the samples could be classified into two molecular subtypes (Figure 8A). Next, PCA results showed significant differences between these two subtypes (Figure 8B). Moreover, DEG analyses also revealed that the expression of SeDGs varied across subtypes (Figure 8C). Clustering analysis of patients can display differences in disease features and biomarkers among subtypes, which can provide clues for the development of new treatments and drugs and advance the development of personalized medicine, thereby providing more effective treatment options for patients.

## 4 Discussion

This study explored changes in peripheral blood gene expression profiles during the complications of sepsis to ALI or ARDS with comprehensive bioinformatics analysis and further evaluated the potential role of cell death-related genes in SeALAR. First, it was observed that immunoregulation-related pathways were substantially altered in the peripheral blood of SeALAR patients, underscoring the importance of immune abnormalities in the development of SeALAR. Through differential gene analysis combined with multiple statistical methods, nine reliable SeDGs were screened and a risk prediction model was constructed based on these genes, which provided a novel means of assessing the clinical prognosis of septic patients. Further immune correlation analysis demonstrated a correlation between SeDGs and the degree of immune cell infiltration in the peripheral blood of SeALAR patients, offering potential targets for personalized immunotherapy.

Among SeDGs, CD19 was highly expressed in the peripheral blood of SeALAR patients and correlated with activated B cells. Generally, CD19 is expressed on the surface of B lymphocytes and is a key molecule in the development and activation of B cells (30). The correlation of CD19 with activated B cells hints at a possible role of B cells in SeALAR by participating in immunoregulation. EXT1 was highly expressed in the peripheral blood of SeALAR patients and was correlated with activated dendritic cells. As reported, EXT1 is a multifunctional glycosyltransferase that assumes a pivotal role in regulating extracellular matrix and cellular signaling (31). The correlation of EXT1 with activated dendritic cells implicates a potential mechanism for mediating dendritic cell function in SeALAR. FEM1B was overexpressed in the peripheral blood of SeALAR patients and was correlated with regulatory T cells. As an E3 ubiquitin ligase, FEM1B is implicated in processes such as cell cycle regulation, signaling, and immunoregulation (32). The correlation of FEM1B with regulatory T cells may indicate that immune responses in SeALAR can be affected by modulating the activity of immunosuppressive cells. As a key regulator of mitochondrial mass and function (33), PINK1 was upregulated in the peripheral blood of SeALAR patients. This gene was correlated with T helper 17 cells, implying that abnormal mitochondrial function is associated with the pathogenesis of SeALAR, which further affects immune cell activity and inflammatory responses. FTH1 was lowly expressed in the peripheral blood of SeALAR patients and was correlated with macrophages. Reportedly, FTH1 is responsible for intracellular storage and release of ferric ions (34, 35), whose correlation with macrophages may underscore the significance of ferric ions in the pathogenesis of SeALAR by modulating immune responses and inflammatory processes. CDKN1A was poorly expressed in the peripheral blood of SeALAR patients and associated with plasmacytoid dendritic cells. CDKN1A not only is a key molecule in cell cycle regulation but also is involved in processes such as DNA damage repair and apoptosis (36). Therefore, its correlation with plasmacytoid dendritic cells may suggest a potential role of cell cycle regulation and apoptosis in the pathogenesis of SeALAR. PI3 was lowly expressed in the peripheral blood of SeALAR patients and was correlated with monocytes. PI3 is a critical signaling regulatory molecule involved in the regulation of various cellular functions (37). Therefore, its correlation with monocytes may illustrate a regulatory role of the PI3 pathway in monocyte-mediated immune responses. PTPRC was expressed at a low level in the peripheral blood of SeALAR patients and was correlated with neutrophils. As PTPRC is a member of the immunoglobulin superfamily that is vital for immune cell signaling (38), its correlation with neutrophils may allude to the pivotal involvement of immune cell activation and inflammatory responses in the pathogenesis of SeALAR. PTGDS was downregulated in the peripheral blood of SeALAR patients and was correlated with activated CD8 T cells. PTGDS is a key synthase for prostaglandin D2 that has been reported to be involved in many physiological and pathological processes (39). Hence, the correlation of PTGDS with activated CD8 T cells may highlight a potential regulatory mechanism of prostaglandin D2 in T cell activity and inflammatory responses during SeALAR. However, the above correlations are potential clues rather than direct evidence. The specific roles of these gene-immune cell axes in the pathogenesis of SeALAR will be explored through functional experiments in combination with cell or animal models in follow-up studies. In addition, a preliminary dissection of the molecular mechanisms and potential subtypes of SeDGs was conducted through GSEA pathway enrichment analysis and clustering analysis. This provided theoretical guidance for the targeted treatment of SeALAR.

Although this study initially constructed a characteristic molecular model for identifying SeALAR patients based on multiple sets of GEO data and cross-validated with multiple machine learning algorithms, there are still several non-negligible limitations. First, all the samples were obtained from two public databases, GSE10474 and GSE32707, with relatively limited sample sizes and possible bias of regions, populations, or sampling platforms. This limits the broad applicability of the findings to some extent. Second, although the internal performance of the prediction model was evaluated by calibration curve and decision curve analysis (DCA), it has not been validated in external multicenter or real-world independent clinical cohorts, and the ability of model of clinical dissemination and generalization still needs to be further tested. Third, the functional inference of differential genes mainly relies on gene set enrichment analysis and signaling pathway annotation, which still lacks experimental evidence at the cellular and animal levels, and thus still needs to be strengthened in mechanism explanation. Fourth, although two molecular subtypes of SeALAR have been successfully identified based on the characterized genes, the differences in clinical phenotype, immune characteristics, disease progression, and therapeutic response between the different subtypes have not been systematically investigated, and the significance of subtyping in precision diagnosis and treatment needs to be further clarified.

To overcome the above deficiencies, future studies can be advanced in the following directions: (1) introduce large-scale, multicenter, and more heterogeneous clinical samples for external validation, and systematically assess the stability, accuracy, and application value of the risk model in different clinical settings; (2) combine the *in vitro* functional experiments (such as gene knockdown or overexpression) and *in vivo* animal models to further validate the specific role and regulatory mechanism of key SeDGs in the SeALAR development; (3) based on the current molecular subtyping, we will explore the differences in immune cell infiltration characteristics, inflammatory factor levels, disease severity, response to interventions, and survival and prognosis of patients with various subtypes, and establish a more clinically instructive subtyping system; and (4) we will integrate the single-cell transcriptomics, proteomics and metabolomics data dynamically and depict the immune microenvironmental changes and signaling pathway remodeling process in SeALAR from a multi-dimensional perspective, providing a solid foundation for the validation of molecular markers and the optimization of target intervention strategies.

In addition, it is worthwhile to further explore the integration of the genetic risk model proposed in this study with existing clinical scoring systems (e.g., SOFA, qSOFA, or APACHE II) to construct a joint prediction model. This can enhance the support of early identification and individualized treatment decision-making for SeALAR patients. If the rapid detection of SeDGs can be realized by combining qPCR or ELISA in the future, it is also expected to promote its translational application to bedside diagnostic tools.

In summary, this study provides a new idea and methodological framework for the molecular mechanism analysis and risk prediction of SeALAR. Through continuous improvement in the biological validation system with larger sample size and higher resolution, we are expected to further promote the translational application of SeDGs in the early identification, clinical typing, and targeted intervention of sepsis-associated ARDS, and to provide a more solid theoretical foundation and practical pathway for precision diagnosis and treatment.

## Data Availability

The original contributions presented in this study are included in this article/Supplementary material, further inquiries can be directed to the corresponding author.
