# Supplementary figures and images for: Cell death-related signature genes: risk-predictive biomarkers and potential therapeutic targets in severe sepsis
Source: Front Med (Lausanne). 2025 May 30;12:1577203. doi: 10.3389/fmed.2025.1577203 (PMC12163320; doi:10.3389/fmed.2025.1577203)

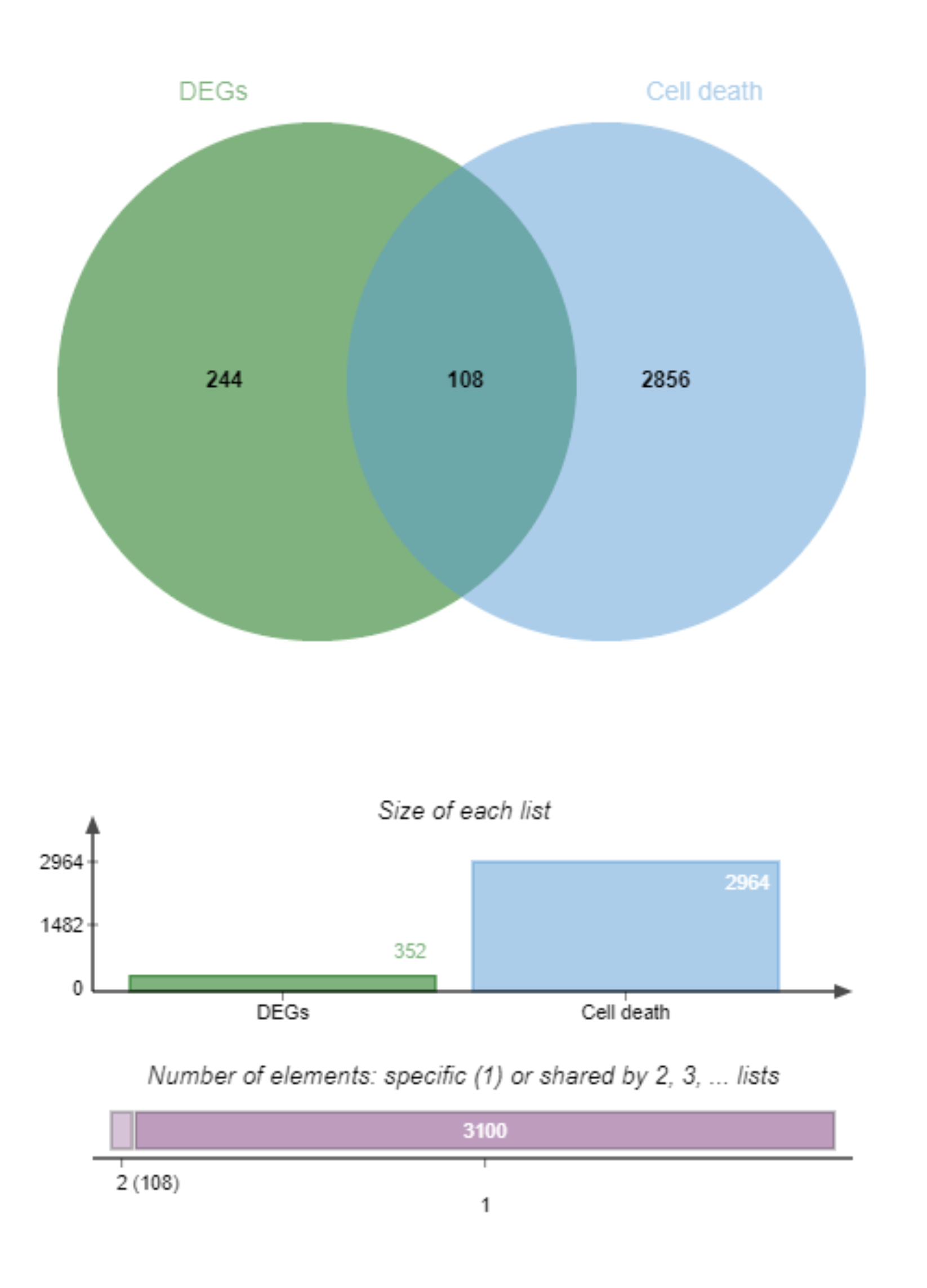

Supplement: Supplementary file 6 [file Image_1.tif]

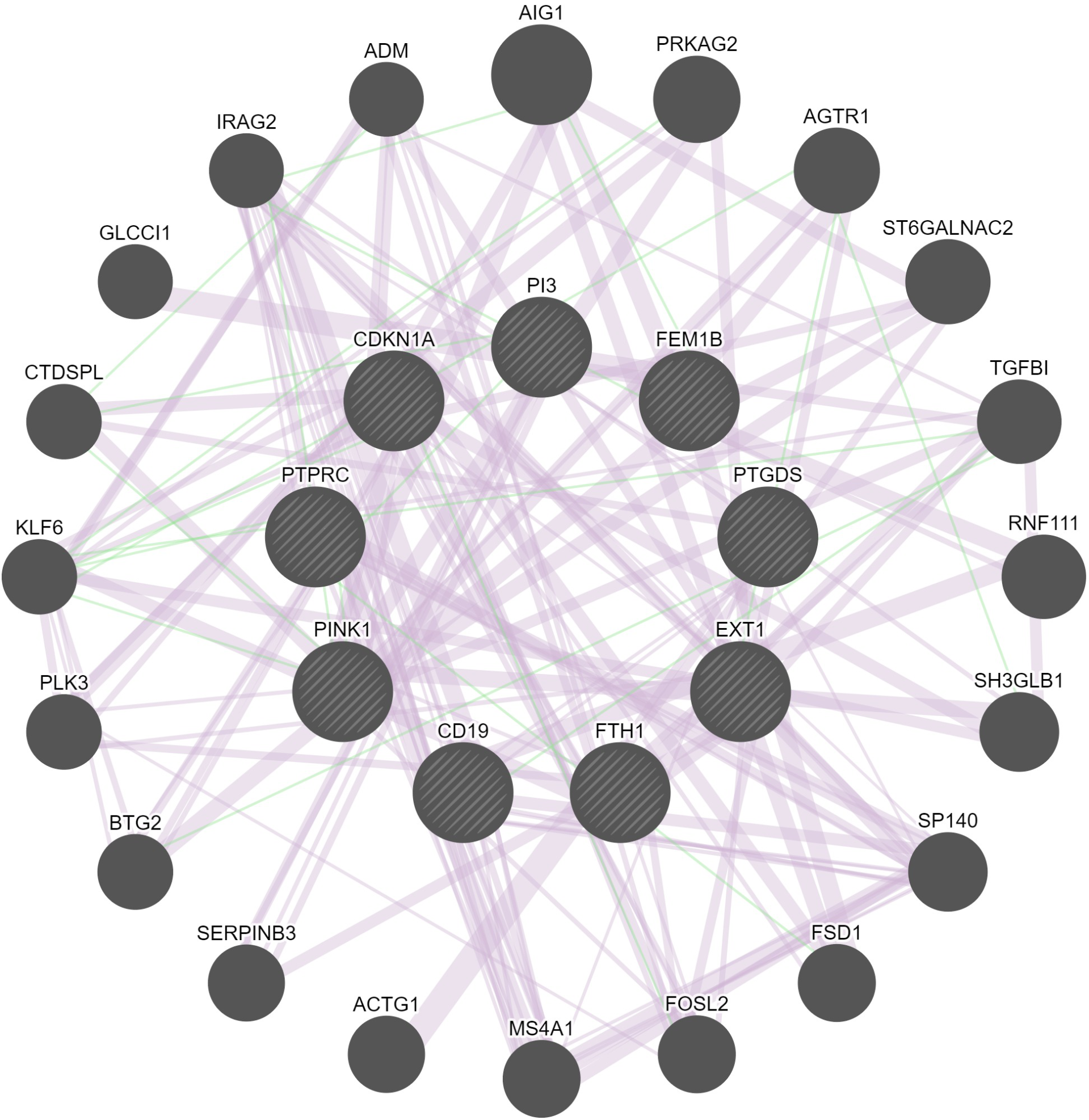

Supplement: Supplementary file 7 [file Image_2.tif]
