# Supplementary material for: Cell death-related signature genes: risk-predictive biomarkers and potential therapeutic targets in severe sepsis
Source: Front Med (Lausanne). 2025 May 30;12:1577203. doi: 10.3389/fmed.2025.1577203 (PMC12163320; doi:10.3389/fmed.2025.1577203)
Supplement: Supplementary file 8 [file Data_Sheet_1.docx]

Supplementary Material

# Supplementary Figures and Tables

## Supplementary Figures

**Figure S1.** Identification of cell death-related genes in SeALAR. Venn diagram showing the intersection of cell death-related genes and DEGs.

**Figure S2.** PPI construction of signature genes in SeALAR.

# Supplementary Tables

**Table·S1** 2856 genes associated with death

**Table S2** Differentially expressed genes in peripheral blood of SeALAR and·Se groups

**Table·S3** 91 upregulated genesand·261·down-regulated genes·in 352-DEGs

**Table S4** Human T-cell leukemia virus type 1 infection, phagosomes, Epstein-Barr virus infection, and Th1 and Th2 cell differentiation in SeALAR

**Table S5** 108 cell death-related genes differentially expressed in SeALAR
